# The Increasing Problem of Resistant Hypertension: We’ll Manage till Help Comes!

**DOI:** 10.3390/medsci12040053

**Published:** 2024-10-04

**Authors:** Francesco Natale, Rosa Franzese, Ettore Luisi, Noemi Mollo, Luigi Marotta, Achille Solimene, Saverio D’Elia, Paolo Golino, Giovanni Cimmino

**Affiliations:** 1 Vanvitelli Cardiology and Intensive Care Unit, Monaldi Hospital, 80131 Naples, Italy; 2 Department of Translational Medical Sciences, Section of Cardiology, University of Campania Luigi Vanvitelli, 80131 Naples, Italy; giovanni.cimmino@unicampania.it; 3 Cardiology Unit, AOU Luigi Vanvitelli, 80138 Naples, Italy

**Keywords:** resistant hypertension, guidelines, cardiovascular mortality, antihypertensive drugs

## Abstract

Arterial hypertension remains the major cardiovascular risk worldwide. It is estimated that under 50 years of age one in every three adults is hypertensive while beyond the age of 50 the prevalence is almost 50% globally. The latest World Health Organization (WHO) Global Report on Hypertension indicated that the global number of hypertensive patients almost doubled in the last three decades, with related increasing deaths, disability, and costs annually. Because of this global increase, early diagnosis and timely treatment is of great importance. However, based on the WHO Global Report, it is estimated that up to 46% of individuals were never diagnosed. Of those diagnosed, less than 50% were on treatment, with nearly half among these at target according to the current guidelines. It is also important to note that an increasing number of hypertensive patients, despite the use of three or more drugs, still do not achieve a blood pressure normalization, thus defining the clinical scenario of resistant hypertension (RH). This condition is associated to a higher risk of hypertension-mediated organ damage and hospitalization due to acute cardiovascular events. Current guidelines recommend a triple combination therapy (renin angiotensin system blocking agent + a thiazide or thiazide-like diuretic + a dihydropyridinic calcium-channel blocker) to all patients with RH. Beta-blockers and mineralocorticoid receptor antagonists, alone or in combination, should be also considered based on concomitant conditions and potential contraindications. Finally, the renal denervation is also proposed in patients with preserved kidney function that remain hypertensive despite the use of maximum tolerated medical treatment. However, the failure of this procedure in the long term and the contraindication in patients with kidney failure is a strong call for a new therapeutic approach. In the present review, we will discuss the pharmacological novelties to come for the management of hypertension and RH in the next future.

## 1. Introduction

Current therapeutic approaches for the management of hypertension (MH), which are mainly based on the use of antihypertensive drugs and lifestyle interventions, have provided notable results in reducing absolute blood pressure (BP) values, in the regression of organ damage, and in the improvement of cardiovascular protection [1,2]. Despite the notable successes of the therapeutic approach achieved applying the current and previous guidelines [1,3,4], numerous areas still require improvement. For example, it has been reported that in hypertensive patients even once the BP is optimized following all the guideline recommendations, a higher risk level still persists compared to normal subjects of the same age [1]. Specifically, the probability that the hypertensive patient will experience a cerebral or cardiovascular event and/or kidney damage remain markedly and significantly higher when compared to normotensive subjects who have never been treated [1]. This phenomenon, defined as “residual risk”, appears to be multifactorial [5], including arterial and organ structural and functional alterations over the years because of hypertension and the difficult achievement of BP control, as recommended by current guidelines [1]. Since the cardiovascular risk (CVR) associated with hypertension (HNT) is strictly and directly related to BP values, the lack of their full normalization can also take part in the phenomenon [1]. Another failure of the pharmacological MH is represented by persistent high blood pressure levels despite the use of three or more full-dose antihypertensive drugs, a condition that is estimated to involve approximately 10% to 15% of treated hypertensive patients [6]. This condition is defined as “resistant hypertension” (RH) and is characterized by a very high CVR profile [6]. RH is defined as BP that remains above the target treatment, despite the use of hypertensive drugs belonging to three or more classes including diuretics. Even patients with controlled BP using four or more classes of medications are considered to have RH [1,2,6,7]. Although the exact prevalence of this phenomenon remains unknown, cross-sectional studies suggest that it includes approximately 10 to 15% of the general hypertension population [6]. The high prevalence of HNT in the general population makes this small percentage very significant in absolute terms. Patients suffering from RH are at high CVR, due to the frequent association with other CVR factors, such as obesity, left ventricular hypertrophy, chronic kidney failure (CKD), and obstructive sleep apnea syndrome [6]. An intensive treatment on top of lifestyle changes is mandatory and guidelines-endorsed in RH patients [2,6,7]. Furthermore, these patients seem to benefit greatly from treatment with mineralocorticoid receptor antagonists (MRAs), especially if renal function is normal [8]. Moreover, patients with RH who, despite maximum tolerated medical treatment, still fail to achieve blood pressure control are defined as suffering from “refractory hypertension” [9]. These observations, together with the intrinsic limits of pharmacological therapy (patient compliance, polypharmacy, and adverse effects), have highlighted the need of other therapeutic options beyond conventional antihypertensive drugs, for which the scientific community is still waiting. In the last two decades, no novel medications for MH have been released. In the present manuscript, we will discuss the potential drugs in development for hypertension and RH treatment.

## 2. Literature Sources and Search Strategy

We performed a non-systematic review of the literature by applying the following search strategy against different electronic databases (MEDLINE, EMBASE, Cochrane Register of Controlled Trials, and Web of Science). Original reports, meta-analyses, and review articles in peer-reviewed journals up to July 2024 regarding hypertension, resistant hypertension, guidelines, and novel pharmacological strategies for HNT were incorporated into the search strategy of the electronic databases. The references of all identified articles were reviewed to look for additional papers of interest to extrapolate the more recent available data on the management of RH.

## 3. International Guidelines with a Long-Lasting Gap

The main strategies currently available for the treatment of HNT consist of lifestyle modifications and modifiable risk factors, combined with drug therapy if necessary.

New 2024 European guidelines have replaced the previous classification of blood pressure which provided for the distinction of hypertension in different degrees and currently recognize only a non-elevated pressure (systolic blood pressure (SBP) <120 mmHg and diastolic blood pressure (DBP) <70 mmHg), a high pressure (SBP between 120 and 139 mmHg and DBP between 70 and 89 mmHg), and hypertension (SBP greater than or equal to 140 mmHg and DBP greater than or equal to 90 mmHg), based on office BP measurements confirmed by out-of-office measurements (home or ambulatory BP monitoring) or at least one repeat office measurement at a subsequent visit [1,4,10]. On the other hand, HNT can be staged based on the presence or absence of hypertension-mediated organ damage (HMOD) as follows:

- Stage 1: Uncomplicated hypertension (i.e., without confirmed HMOD or cardiovascular disease (CVD), but including stages 1 and 2 of CKD);
- Stage 2: Presence of HMOD or CKD stage 3 or diabetes;
- Stage 3: Established cardiovascular disease (CVD) or CKD stage 4 or 5.

Due to the high prevalence of hypertension in the general population and its significant role as a cause of morbidity and mortality, opportunistic screening is recommended for all adult individuals starting at age 18 [1]. The previous European guidelines recommended to immediately start pharmacological treatment in all patients with grade 2 and 3 HNT, while for patients with grade 1 HNT only if they had a high CVR. In those with grade 1 hypertension in the absence of high CVR, pharmacological therapy was recommended only if lifestyle changes did not achieve a sufficient result. In the new guidelines, there have been important changes in recommendations. According to current guidelines, it is recommended to start drug treatment immediately in all patients with HNT irrespective of CVR and to associate lifestyle changes in all patients to obtain better blood pressure control and a reduction in CVR [4]. Initiation of drug therapy is recommended in patients with elevated pressure who have a sufficiently high CVR [4] and a confirmed BP of ≥130/80 mmHg after three months of BP-lowering lifestyle measures when these lifestyle changes have not worked. A sufficiently high CVR is described as established CVD (atherosclerotic cardiovascular disease or heart failure) or moderate–severe CKD or other forms of HMOD (left ventricular hypertrophy or atheromasia of the supra-aortic trunks or coronary artery calcium score >100 Agatston units or peripheral artery disease) or diabetes type 1 or 2 or probable or certain familial hypercholesterolemia or in presence of SCORE-2 or SCORE-2 OP at least 10% [4] or a borderline score (5–10%) with additional risk factor modifiers. On the other hand, patients with elevated pressure who do not have a high CVR are recommended for lifestyle modifications; associated drug therapy might be discussed on an individual basis when despite these modifications at 6–12 months BP is between 130/80 and <140/90 mmHg [4]. The major limitations of all guidelines published in the last 15 years remain the lack of novelty in pharmacological approaches with basic and clinical research in this field still stopped.

### 3.1. “Let Food be Thy Medicine and Medicine be thy Food”

This famous sentence spoken by Hippocrates around 400 BC has never been more relevant for CVD.

The lifestyle modifications currently recommended by the HNT guidelines are:

- body weight reduction: for every kilogram of weight loss, a reduction in SBP and DBP of approximately 1 mmHg is estimated [11]. To this end, a low-calorie diet is recommended [12]. Among the various diets, those associated with a greater reduction in BP are the Dietary Approaches to Stop Hypertension (DASH diet) [11] and the Mediterranean diet [12]. The new ESC guidelines on HNT recommend a stable BMI of 20–25 kg/m^2^ and a waist circumference less than 94 cm in males and less than 80 cm in females [4];
- reducing dietary sodium intake: a daily sodium intake <100 mmol (5.8 g of salt per day) has been shown to be associated with an average reduction of about 5 mmHg in SBP and 2 mmHg in DBP in patients with HNT [13]. Salt (NaCl) restriction to <5 g (~2 g sodium) per day as well as the use of salt substitutes, which have also shown reductions in SBP of about −4.8 mmHg and DBP of about −1.5 mmHg, is recommended [1,14];
- increased dietary potassium intake: administration of 60 mmol (1380 mg) of potassium chloride has been observed to reduce BP by approximately 2 and 4–5 mmHg in adults with normotension and HNT, respectively [15]. The ESC guidelines recommend, in class IIb, in patients who do not have moderate to severe renal insufficiency, to increase potassium intake by 0.5–1 g/day either by replacing sodium with potassium-enriched salts or through a diet rich in fruit and vegetables [4];
- regular physical activity: aerobic exercise is associated with an average reduction in SBP of approximately 2–4 and 5–8 mmHg in adult patients with normotension and hypertension, respectively [16]. New ESC guidelines recommend moderate-intensity aerobic exercise (40–60% heart rate reserve) for at least 150 min per week, or 75 min per week of vigorous-intensity aerobic exercise for three days associated with low- or moderate-intensity dynamic or isometric resistance training (2–3 times/week) to prevent and treat HNT and CVD [1,4,17];
- reduction in alcohol intake: A major meta-analysis revealed that reducing alcohol intake close to abstinence in people who habitually consumed alcohol (at least three drinks/day) was associated with a reduction of 3.3 mmHg in SBP and 2.0 mmHg in DBP [18]. The guidelines are not unambiguously clear on maximum limits, but recommend moderating alcohol intake [1]. New ESC guidelines recommend preferably avoiding alcohol intake or in any case drinking less alcohol than the maximum limit, which is about 100 g/week of pure alcohol [4];
- abstention from cigarette smoking: compared to non-smokers, smokers more frequently present with malignant hypertension, documented by normal ambulatory BP values and higher daytime BP values at 24 h ambulatory BP monitoring [19] and very variable as smoking causes a sympathetic activation that is associated with an increase in BP for about 30 min [20].

A new recommendation from the new 2024 ESC guidelines on HNT concerns avoiding sugary drinks from a young age and reducing free sugar intake in the diet to less than 10% of daily energy intake [4].

It is important to point out that across the guidelines from different international societies lifestyle adjustments are different and it is unclear which ones are most effective [21].

A 2020 meta-analysis that included 126 RCTs found that of 22 non-pharmacological interventions compared with “usual care” (i.e., patients who continued to lead their lifestyle without making changes), the DASH diet was the most effective strategy in reducing BP even alone in patients with prehypertension and in those with HTN. The meta-analysis demonstrated with moderate or high quality of evidence a moderate efficacy of some other non-pharmacological interventions such as the aforementioned aerobic exercise, reduced sodium intake, increased potassium intake, and alcohol restriction, but also a series of holistic interventions such as yoga (sessions of at least 30 min three times a week), meditation (20 min twice a day sitting in a comfortable position), or breathing control (breath control sessions for a total of 40 min a week with the aim of reducing the respiratory rate to less than 10 breaths/minute). Traditional Chinese exercises such as tai chi (three times a week) or qigong (twice a week) also seemed to have a favorable efficacy profile, but the evidence in this regard was of low quality. Further randomized trials are needed to ensure that they fall within the recommendations of the guidelines [12].

**Figure 1:**
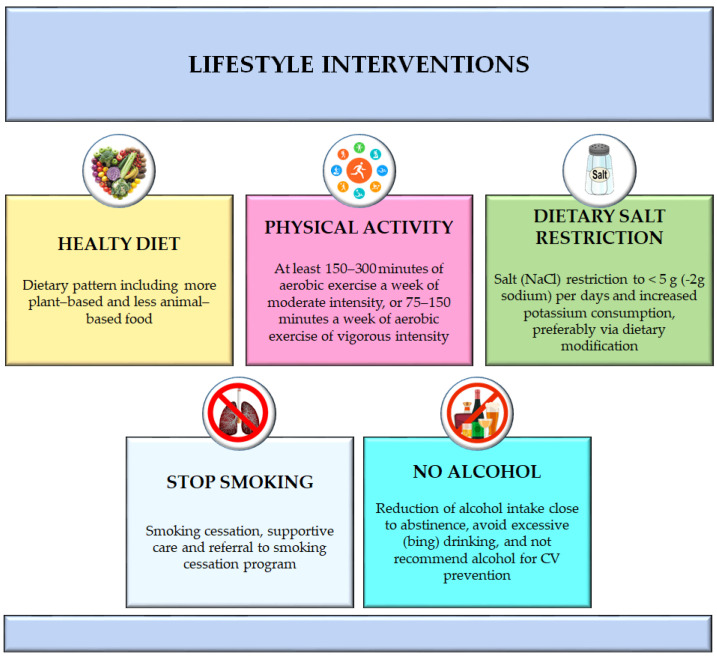
Lifestyle recommendations: current HNT guidelines summarized regarding lifestyle interventions as a healthy diet, regular physical activity, dietary salt restriction, reduction in alcohol intake, and smoking cessation.

A schematic view of lifestyle interventions is summarized in Figure 1.

### 3.2. Combination Strategy as First: When Two Are Better than One

With regard to pharmacological treatment, ESC and ESH guidelines recommended five main classes of drugs as first-line pharmacological treatment for HNT: ACE inhibitors (ACEis), angiotensin receptor blockers (ARBs), calcium antagonists (CCBs), thiazide or thiazide-like diuretics, and beta blockers (BBks) [10]. New 2024 ESC guidelines on HNT recommend as first line ACE inhibitors (ACEis), angiotensin receptor blockers (ARBs), calcium antagonists (CCBs), and thiazide or thiazide-like diuretics because they have proven to be more effective [4].

The guidelines recommended a treatment algorithm that promotes combination therapy as the first line of drug treatment in most patients [4] as it would appear to reduce the problem of therapeutic inertia [22], improve adherence to treatment, and improve BP control [23,24]. Monotherapy may only be considered in the setting of moderate-to-severe frailty, limited life expectancy, symptomatic orthostatic hypotension, or older people (aged ≥85 years), or in patients with elevated BP and increased CVR [1,4].

Basically, all major drug classes can be combined, except for ACEis and ARBs, since this combination is associated to a risk of worsening renal function (40%), hyperkalemia (40%), and hypotension (42%) [25]. Guidelines recommend starting treatment preferentially with a combination of ACEis with CCBs or ACEis with thiazide/thiazide-like diuretics. In fact, studies that have compared two different drug combinations head-to-head in a randomized manner are few. The ACHIEVE study has shown that despite no significant differences in blood pressure reduction, the ACEi or ARB + CCB combination was superior to the same ACEi or ARB + thiazide diuretic combination in preventing cardiovascular outcomes and chronic kidney disease progression [26]. On the other hand, the COLM and COPE studies have reported no significant differences in cardiovascular events in the arm receiving the ACEi/ARB–CCB blocker combination compared to that receiving the thiazide ACEi/ARB–diuretic blocker combination [27,28].

These combinations are available in a single-pill formulation with a wide range of doses, as per guideline recommendations, to facilitate adherence to treatment and titration of therapy, limit adverse effects associated with high doses of certain drug classes, and improve the speed and efficacy of therapy [1]. If combination therapy with two drugs (titrated to the maximum tolerated dosage) is not sufficient to achieve the desired BP target, the guidelines recommend a single-pill therapy consisting of three drugs: an ACEi or ARB in combination with CCB and thiazide or a thiazide-like diuretic [1,4].

**Figure 2:**
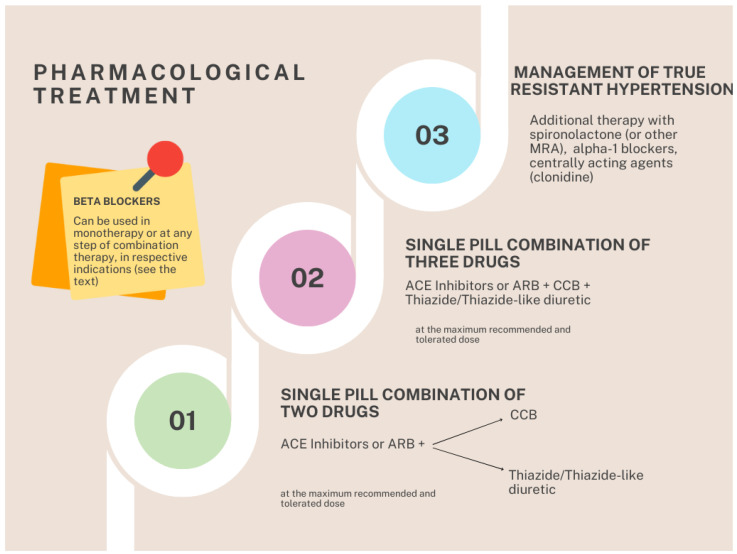
Pharmacologic treatment and combination strategies. First line, a variable combination of two classes of drugs (ACEi or ARB associated with a CCB or a thiazide or thiazide-like diuretic), preferably in one single-pill administration. Second line, adding the one of these drugs that the patient is not taking yet, preferably in one single-pill combination. Third line, adding spironolactone or if not effective or tolerated eplerenone instead of spironolactone and next adding alpha 1 blockers or centrally acting agents like clonidine or one of the others potassium-sparing diuretics. BBks are recommended at any step in monotherapy or as combination therapy only in patients with acute and chronic coronary syndrome; arrhythmias; heart failure with reduced or preserved ejection fraction with ischemic etiology; atrial fibrillation; women of childbearing age who wish to become pregnant; HNT during pregnancy. ACE: angiotensin-converting enzyme (ACE), ARB: angiotensin receptor blocker, CCB: calcium channel blocker, MRA: mineralocorticoid receptor antagonist.

A summary of these combination strategies is shown in Figure 2.

The use of renin-angiotensin system (RAS) inhibitors is considered the common denominator of the combination treatment strategy. ACEis are the most widely used antihypertensive drug and are recommended mainly in patients with CAD or heart failure (HF) as they are also associated with protective effects in terms of cardiovascular events; they are, however, contraindicated in patients with severe hyperkalemia (>5.5 mmol/L), bilateral renal artery stenosis, pregnant women, or those of childbearing age. ACEis can cause a cough in 5–10% of subjects and more rarely chronic angioedema, side effects that are much more infrequent with ARBs [1].

CCBs are particularly effective in patients of African descent and in the older patient population. The use of non-dihydropyridine CCBs is not recommended in patients with severely reduced ejection fraction (ejection fraction EF <40%), atrio-ventricular or atrial sinus blocks of any degree, or bradycardia due to their pronounced negative inotropic effect [1].

Thiazide/thiazide-like diuretics are not only effective as antihypertensives but are also involved in cardiovascular prevention. Both thiazide and thiazide-like diuretics may cause hypokalemia and, rarely, gout attacks. They are contraindicated in patients with hyponatremia and in patients with renal disease due to obstructive uropathy [1]. They are less effective in patients with advanced CKD (eGFR < 30 mL/min/1.73 m^2^) for whom guidelines recommend choosing loop diuretics (furosemide, torasemide, bumetanide) as diuretics for the treatment of HNT, which are generally not recommended in the treatment of uncomplicated HNT. The choice of loop diuretics in favor of thiazide diuretics may also be useful in patients with significant fluid retention, such as those with HF [1].

The combination of CCBs and diuretics should be closely monitored because of reported adverse effects [29]. Beta-blockers can be used as monotherapy or combination therapy, at any step, in hypertensive patients with acute and chronic coronary syndrome; arrhythmias; heart failure with reduced or preserved ejection fraction with ischemic etiology; atrial fibrillation; women of childbearing age who wish to become pregnant; HNT during pregnancy; excessive pressure response to exercise and stress [1]. This class of drugs is contraindicated in patients with severe asthma, atrioventricular or atrial sinus blocks of any degree, and bradycardia. They have a less favorable side-effect profile than RAS inhibitors, although third-generation BBks have fewer side effects [30]. They are equivalent to the other drug classes in the prevention of cardiovascular events except for stroke [31,32]. If BP is not controlled despite triple combination therapy (at the highest recommended tolerated dosage), it must be treated according to the recommendations of the guidelines for true RH [1,10].

Potassium-sparing anti-aldosteronic diuretics, first of all spironolactone, must be added if blood pressure is not controlled by triple combinations of the main drug classes.If spironolactone is not tolerated or it is not enough to control BP, consider adding eplerenone in place of spironolactone or a beta blocker, if not already indicated, and only then a centrally acting drug such as alpha-blockers, alpha metildopa or clonidine, angiotensin/neprilysin receptor inhibitors (ARNIs), or other potassium-sparing diuretics [4,10].

Alpha 1 blockers are drugs found in some studies to be effective in preventing incident or fatal CAD, but have been associated with a higher incidence of HF [33], and also found to be less effective than spironolactone as third-line therapy in controlling RH [34]. These drugs can cause orthostatic hypotension and water retention. MRAs are used exclusively in hypertensive patients with hyperaldosteronism or RH. The absence of data on their effect on preventing cardiovascular outcomes in hypertensive patients and the risk of hyperkalemia has probably limited their routine use in the treatment of HNT. For this purpose, new non-steroidal MRAs such as finerenone are currently under investigation [35]. ARNIs are currently not approved for the treatment of HNT in Europe and the United States of America [1], although they have been shown to be more effective in reducing blood pressure than other drugs such as valsartan, olmesartan, and amlodipine [36]. They are currently used in clinical practice to treat patients with HF [37].

Centrally acting drugs such as alpha-methyldopa or clonidine are becoming less and less recommended as routine therapy in HNT due to reduced evidence of their protective effects on cardiovascular outcomes in hypertensive patients and their poor tolerability profile compared to other drugs. For this reason, these drugs are now reserved for combination therapy in the treatment of RH in which other drugs have not given the expected effects [1]. Vasodilators such as nitrates and nitroprusside are very effective in reducing arterial BP, but are now mainly used for the intravenous treatment of hypertensive emergencies [1].

### 3.3. Special Population

Specific attention should be reserved to define classes of patients:

Young adults: in patients aged <40 years, it is first indicated to search for causes of secondary hypertension and, if present, treat them. The algorithm suggested by the guidelines is not faithfully applicable since SCORE-2 is not validated in these patients. Therefore, the guidelines recommend starting treatment in those patients with BP >140/90 mmHg or in patients with elevated BP who have a high cardiovascular risk (established CVD, diabetes mellitus, familial hypercholesterolemia, and moderate or severe CKD or who have organ damage at HMOD assessment) [4]. Regardless of CVR, in all young patients with high BP it is recommended to follow lifestyle changes BP lowering [4].

Elderly: in patients aged < 85 years who are not moderately or severely frail, the guidelines tell us to use the same recommendations we use in the general population. The guidelines tell us that antihypertensive treatment can be continued for life if adequately tolerated. In patients aged 85 years or older, moderately or severely frail, with pre-treatment orthostatic hypotension and/or limited life expectancy, the guidelines recommend considering treatment only if BP is greater than or equal to 140/90 mmHg and to start treatment preferring CCBs or RAS inhibitors [4].

Chronic kidney disease: in patients with moderate or severe chronic kidney disease (eGFR <60 mL/min/1.73 m^2^) and high blood pressure, the guidelines recommend starting antihypertensive treatment with drugs and lifestyle changes. They emphasize that among the former, drugs such as CCBs or RAS inhibitors should be preferred and that patients with an eGFR < 20 mL/min/1.73 m^2^ should also consider sodium-glucose co-transporter 2 inhibitors. Among lifestyle changes, however, they recommend reducing sodium intake while avoiding the use of potassium-enriched salts, which should be used with caution in these patients [4] (https://doi.org/10.1093/eurheartj/ehae178). 

Liver failure: The management of patients with liver failure requires a careful, personalized, and integrated approach between cardiology and hepatology specialists to optimize treatment and follow-up. It is crucial to closely monitor the tolerance to antihypertensive drugs, as impaired liver function can affect the response to treatment and the risk of side effects. On the other hand, the use of non-selective beta-blockers, such as propranolol and carvedilol, remains a key strategy to reduce portal pressure and prevent complications like esophageal variceal bleeding [38].

### 3.4. Hypertension-Mediated Organ Damage: Determinants of Aggressive Strategy

Assessing HMOD during the clinical evaluation for all patients with HNT is mandatory since established HMOD impacts on cardiovascular outcome [1]. HMOD assessment is also recommended during follow-up because HNT is a progressive disease, and organ damage may develop subsequently, increasing CVR [39]. On the contrary, a reduction in HMOD serves as an indicator of treatment success. It is important to point out that some types of HMOD can be reversed by antihypertensive therapy, especially if initiated early. However, with long-standing HNT, HMOD may become irreversible despite blood pressure control [40].

One of the most affected organs is the heart; increased afterload leads to structural and functional changes responsible for the development of hypertensive heart disease, which includes the development of myocardial hypertrophy (LVH), geometric alterations of the left ventricle (LV), impaired diastolic and systolic LV function, left atrial dilation, and an increased incidence of arrhythmias [41].

HNT is the second leading cause, after diabetes mellitus, of CKD development in the general population [41]. Impaired renal function can be assessed by estimating the glomerular filtration rate (eGFR) from serum creatinine levels. Hypertensive nephrosclerosis is considered HMOD, which diagnosis is based on reduced renal function or the detection of albuminuria [1]. The albumin-to-creatinine ratio (ACR) is measured from a spot urine sample and is the preferred method for quantifying urinary albumin excretion. Another parameter to evaluate using spectral Doppler ultrasound is the renal resistive index (RRI), a noninvasive and reproducible measure that investigates arterial compliance and/or resistance. Elevated RRI is associated with subclinical signs of renal organ damage in hypertensive patients with normal renal function [42].

At the ocular level, HNT, by damaging retinal vessels, is responsible for the onset of hypertensive retinopathy finally leading to blindness. Other retinal vascular pathologies include retinal vein and artery occlusion and ischemic optic neuropathy [43].

HNT is a significant risk factor for acute cerebrovascular events such as ischemic stroke, intracranial hemorrhage, and transient ischemic attacks (TIAs). Additionally, hypertension leads to pathological changes in cerebral microvessels, with progressive occlusion of small arteries supplying subcortical brain areas, resulting in cerebral microhemorrhages and lacunar infarcts [43]. Brain atrophy and white matter lesions are also associated to HNT and linked to increased risk of cognitive impairment [44].

Finally, HNT is responsible for an increase in the stiffness of large arteries [45]. The gold standard in Europe for measurement is represented by carotid–femoral pulse wave velocity [10,46]. Assessing stiffness can be clinically useful in hypertensive patients, as its elevation is highly prevalent in the hypertensive population [46].

For evaluating HNT-related peripheral arteriopathy, screening measures include the ankle-brachial index (ABI) and assessment of carotid intima-media thickness (IMT) [10].

Although the international recommendation published as guidelines are periodically revised, in the last two decades a huge gap developed in terms of pharmacological research. Since the latest approval of aliskiren in 2007 by the FDA and 2009 by the EMA, no more antihypertensive drugs have been approved to date for HNT. However, research is ongoing and development of new drugs is on the way. A more comprehensive approach to new drugs is expected in the forthcoming new guidelines.

## 4. Resistant Hypertension: the Dark Side of the Moon

Resistant hypertension is a particular form of HNT characterized by SBP ≥ 140 mmHg and DBP ≥ 90 mmHg despite lifestyle measures and optimized medical treatment (OMT), at maximum tolerated doses [6]. OMT must include at least three drugs, one of which should be a diuretic, with an ACEi or ARB and a CCB [1,10]. However, according to the American consensus statement on RH, this condition can be defined as the concurrent use of four or more antihypertensive drugs irrespective of BP levels [47]. Table 1 reports the definition according to ESC and AHA guidelines.

Furthermore, refractory hypertension is a particular form of RH, which is characterized by persistent hypertension after the assumption of five or more drugs of different classes, including a long-acting thiazide-type diuretic and an MRA [47].

It is estimated that RH affects 5–30% of hypertensive patients under treatment, determining an important economic burden on the health care system worldwide [6], but excluding patients with pseudo-RH and applying more selective criteria, the true prevalence decreases to <10% [1,10].

RH is associated with a higher risk of HMOD, non-alcoholic fatty liver disease (NAFLD), CKD, cognitive impairment, dementia for white matter lesions, and premature cardiovascular events, which are not registered in patients with pseudo-hypertension [6,9,48]. The typical patient affected by RH is characterized by older age (>75), male sex, black African origin, obesity, diabetes, atherosclerotic disease, CKD, high sodium intake, high baseline BP, left ventricular hypertrophy, and HF [1,48,49].

Pseudo-RH can be related to poor therapy adherence (≤50% of patients), white coat hypertension (when office BP is higher than BP measured at home or by ambulatory blood pressure monitoring), office BP measurement mistakes, brachial artery calcification, and therapeutical inertia (insufficient drug doses or incorrect associations) [1,10].

**Figure 3:**
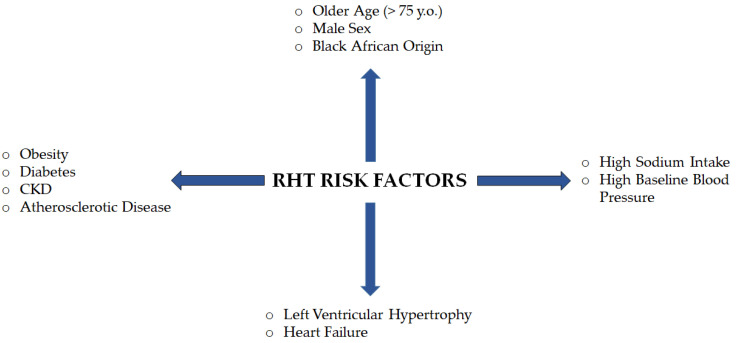
Risk factors for RH (older age, male sex, black African origin, obesity, diabetes, CKD, atherosclerotic disease, left ventricular hypertrophy, heart failure, high sodium intake, high baseline blood pressure).

A schematic view of risk factors for resistant hypertension is reported in Figure 3.

Clinical history analysis is the first step to diagnose this condition: lifestyle, alcohol or sodium intake, drugs or substances consumption, and sleep history should be investigated. The diagnosis of resistant hypertension should be confirmed by physical examination, laboratory tests, and ambulatory blood pressure monitoring (ABPM), which is considered the gold standard according to the ESH, or home blood pressure monitoring (HBPM) [47,49]. Furthermore, all causes of pseudo-resistant hypertension and secondary hypertension should be excluded [1].

### 4.1. Pseudo-RH: Drug Adherence

Inadequate adherence to antihypertensive therapy is one of the most important causes of pseudo-RH [50]. It is characterized by an irregular drug intake and/or early treatment discontinuation [50]. According to the latest ESC guidelines, it correlates with a higher risk of CVD events [4]. A recent study indicates that poor drug adherence is the main type of pseudo-RH in patients who are candidates for renal denervation [51]. Number of pills, low level of education, younger age, and female sex are the main reasons for non-adherence; psychological factors (e.g., fear of adverse events) also play an important role [50]. Evaluation of drug adherence is very difficult; ESC guidelines suggest either detecting prescribed drugs in blood or urine samples or directly observing pill intake during ABPM [4]. Many questionnaires are being used to evaluate adherence such as the Moriski Medication Adherence Questionnaire (MAQ) [52]. However, questionnaires are not recommended [52]. ESC guidelines suggest improving patient–physician communication skills, choosing long-acting drugs, and single-pill combinations as effective methods to improve adherence [4,50]. Modern solutions, such as smartphone apps supported by personalized medical advice, are very promising [53].

### 4.2. Secondary Hypertension

According to more recent ESC guidelines, at least 50% of patients with suspected RH are, instead, affected by secondary hypertension. Primary aldosteronism is the most common cause, especially observed in patients with BP >180/110 mmHg, which can be screened by the aldosterone-to-renin ratio (ARR). Regardless of BP levels, it is associated with higher CVD risk. Renovascular hypertension is characterized by raised BP due to activation of RAAS related to renal artery stenosis or occlusion. Atherosclerosis and fibromuscular dysplasia are the most common causes. It can be suspected by elevated renin levels. Imaging tests include renal doppler ultrasound, abdominal CT angiography, and magnetic resonance imaging (MRI).

Obstructive sleep apnea syndrome can be suspected in obese patients with dipping or reverse-dipping patterns under 24 h BP monitoring. Final diagnosis is confirmed by polysomnogram. Another important cause is phaeochromocytoma which is characterized by high blood levels of catecholamines.

Finally, the use of different drugs can determine iatrogenic secondary hypertension. Among those, it is important to cite non-steroidal anti-inflammatory drugs (which can also reduce BP-lowering drugs’ efficacy), sympathomimetic drugs, and also liquorice [4].

### 4.3. Renal Denervation

Catheter-based renal denervation represents a non-pharmacological therapeutic option for the treatment of HNT, involving the interruption of afferent and efferent sympathetic nerves in the adventitia and perivascular tissue of the renal arteries [54]. Recent controlled clinical studies have demonstrated the efficacy of this procedure in reducing BP, with or without concomitant antihypertensive medications [55,56]. Moreover, the BP reduction effect may be sustained over the years [57], making this technique an interesting approach for patients with suboptimal medication adherence.

The procedure is relatively safe, with a complication rate of renal artery stenosis/dissection of 0.25–0.5% [58]; additionally, no deterioration in renal function has been observed during follow-up [59]. However, several concerns warrant attention. Firstly, the BP reduction effect appears to be relatively modest [60], meaning that adults undergoing this procedure may still require pharmacological treatment. Secondly, the potentially continuous effect could backfire if complications arise, unlike pharmacological therapy, which can be discontinued at any time. Finally, there are no outcome studies demonstrating that renal denervation reduces cardiovascular events, and long-term safety data are lacking. Based on the available evidence, the latest European guidelines suggest that renal denervation may be considered in patients with RH not controlled with a combination of antihypertensive medications, who express a preference for undergoing the procedure after being adequately informed [4]. On the other hand, it may be considered in patients with an increased CVR and uncontrolled HTN on fewer than three drugs due to intolerance or non-compliance, following a shared decision-making process.

## 5. Antihypertensive Drug Development: Research Must Go on

For years, physicians lacked novel pharmacological approaches to use in regular practice and the guidelines periodically revised in the last two decades have limited the “new” recommendations to single-pill combination therapy as first line choice [10], defining a stepwise approach [10] as well as a new grade of HNT such as diastolic hypertension [1]. Because of the recent advancement in antihypertensive drugs, a more comprehensive and up-to-date approach to new drugs is expected from the new ESC and AHA guidelines to come soon. This new pharmacological approach will be addressed in detail.

### 5.1. Finerenone

Hyperaldosteronism is involved in the pathogenesis of systemic HNT and in particular RH. A previous study enrolling 251 patients showed that daytime, night-time, and 24 h SBP and DBP were significantly higher in patients with higher blood aldosterone levels [61]. For this reason, MRAs, including spironolactone and eplerenone, have been recommended as a therapeutic option for the treatment of arterial hypertension. Spironolactone has demonstrated clear efficacy in the treatment of RH and was therefore added as the fourth therapeutic option by the US guidelines for HNT [3]. However, some side effects such as gynecomastia and hyperkalemia limit its regular use. Eplerenone, a selective MRA, has less efficacy as an antihypertensive drug and, like spironolactone, can induce increases in blood potassium levels. Finerenone is a new selective nonsteroidal MRA (nsMRA) with a high affinity and specificity that acts as an inverse agonist by blocking the effects of aldosterone and inhibiting co-regulator recruitment even in the absence of aldosterone. Spironolactone and eplerenone instead appear to act as partial agonists, resulting in some level of co-regulator recruitment at high concentrations [62]. As shown by an animal model study, finerenone exerts a greater effect than eplerenone in improving myocardial contractility, with antifibrotic properties almost absent with eplerenone. These findings could be determined by the inhibition of profibrotic TNX gene expression mediated by differential MR cofactor binding [63]. It has also been demonstrated that finerenone is able to determine a lower increase in pressure overload-induced cardiac left ventricular mass compared to eplerenone, probably due to a differential cardiac gene expression, including differential expression of BNP (brain natriuretic peptide) and Tnt2 (troponin T type 2) [64]. The FIDELIO-DKD trial analyzed the efficacy of finerenone in improving renal function in 5734 patients with CKD and type 2 diabetes mellitus (T2DM) followed for a median follow-up of 2.6 years. Specifically, the relationship between office SBP and the effect of finerenone on cardiorenal outcomes was analyzed. The use of finerenone resulted in a reduction in kidney disease progression. These benefits were achieved by adding finerenone to standard therapy with ACEis or ARBs, in patients with good glycemic and blood pressure control, without recording an excess of adverse events [65]. The protective effect on kidney function occurred independently of baseline office SBP. The mechanisms underlying finerenone benefits on kidney and cardiovascular system in patients with CKD and T2DM could be of hemodynamic and non-hemodynamic nature [35]. The FIGARO-DKD trial analyzed specifically the cardiovascular effects of finerenone. It enrolled 7437 patients from 48 countries with T2DM and mild to moderate CKD receiving therapy with optimized RAS inhibitors. They were randomized, in a 1:1 ratio, to oral therapy with finerenone (10 or 20 mg once daily) or to placebo. The primary endpoint was a composite of time to death from CVD, nonfatal myocardial infarction, nonfatal stroke, or hospitalization for HF. The median follow-up was 3.4 years. The relative risk of the endpoint was significantly reduced by 13% with finerenone compared to placebo. The renal protective effect was also greater than with placebo. However, FIGARO-DKD also demonstrated a weak effect of finerenone on reduction in blood pressure [66]. Few data exist on the hemodynamic effects of finerenone in patients with CKD and BP. ARTS-DN was a multicenter, randomized, double-blind, placebo-controlled, parallel group, phase 2b study designed to analyze and compare the efficacy and safety of finerenone administered at doses of 1.25–20 mg once daily vs. placebo when added to standard of care with a RAS blockers in patients with CKD and T2DM on 24 h ABPM and to evaluate its circadian profile. A total of 821 patients were randomized and treated with either finerenone or placebo. ABPM was performed in 240 of the enrolled patients. The trial demonstrated that finerenone administration had a modest and non-significant effect in reducing office BP from baseline. In contrast, 24 h ABPM showed SBP was significantly reduced from baseline with finerenone. Compared with placebo, finerenone also reduced daytime and night-time SBP. Renal epithelial mineralocorticoid receptors modulate salt and volume handling and consequently blood pressure regulation by the kidneys. Moreover, they also modulate BP through effects on endothelial function. Renal protection may be determined by reduction in BP load on the vasculature. Finerenone has a short plasma half-life of approximately 2–3 h and has no active metabolites and was administered once daily in the morning. So, it is likely that the reduction in BP over 24 h with finerenone was not only produced by its pharmacokinetic effects but maybe by transcriptional effects [67].

Safety and BP-lowering efficacy of spironolactone and finerenone were compared in the FIDELITY trial (a pooled analysis of FIDELIO-DKD and FIGARO-DKD) that identified a subgroup of patients with RH and CKD meeting the eligibility criteria of the AMBER trial which evaluated the effectiveness of the addition of patiromer to spironolactone in the treatment of RH in CKD. The main outcomes were mean change in SBP, incidence of potassium blood levels ≥5.5 mmol/L, and hyperkalemia-associated treatment discontinuation. FIDELITY results at 17 weeks were compared with 12 weeks from AMBER, showing that finerenone was associated with a lower risk of hyperkalemia and treatment discontinuation as well as to a lower SBP reduction [68].

In conclusion, traditional MRAs appear to present a greater BP-lowering effect compared to finerenone. However, the cardioprotective and renoprotective effects of finerenone and lower risk of hyperkalemia are significant. Further studies on a larger number of patients are needed to evaluate the efficacy of finerenone on RH [69].

### 5.2. Baxdrostat and Lorundrostat

Another therapeutic option for the treatment of RH is represented by aldosterone synthase inhibitors, in particular baxdrostat and lorundrostat. Compared to spironolactone and eplerenone, these drugs act not through aldosterone receptor blockade but through selective enzymatic inhibition of aldosterone synthases. Furthermore, they have been shown to block both the genomic and non-genomic effects of aldosterone [70]. Aldosterone synthase produces aldosterone through three steps starting from its precursor, 11-deoxycorticosterone. The first drug in this category was osilodrostat. A first study comparing its efficacy vs. eplerenone demonstrated a similar reduction in SBP levels [71], while in a subsequent trial enrolling 155 RH patients a lower reduction was reported [72]. Aldosterone synthase (encoded by the CYP11B2 gene) shares 93% sequence similarity to 11β-hydroxylase (encoded by the CYP11B1 gene). This would also cause a reduction in the synthesis of cortisol and consequently an increase in the release of ACTH by the adenohypophysis which would stimulate the adrenal cortex to produce 11-deoxycorticosterone as a compensation mechanism. This would reduce the effectiveness of blocking aldosterone synthase. For this reason, new drugs have been studied that act on the same target, but with a major selectivity. These include baxdrostat, which shows approximately 100-fold greater selectivity for CYP11B2 than for CYP11B1. In preclinical and early clinical studies, it has also been shown to suppress aldosterone production completely in humans without affecting cortisol production [73]. The BRIGHT-HTN trial was a multicenter, randomized, double-blind, phase 2 trial that enrolled 275 RH patients, with BP levels > 130/80 mmHg, already on therapy with antihypertensive drugs of three different classes, including a diuretic. They were randomized to receive baxdrostat at a dosage of 0.5 mg, 1 mg, 2 mg once daily for 12 weeks, or placebo. The primary endpoint was the degree of reduction in SBP levels after 12 weeks of therapy. The secondary efficacy endpoint was instead represented by the reduction in DBP and the percentage of patients who reached the blood pressure target of less than 130/80 mmHg at 12 weeks. A total of 248 patients completed the study. At the end of 12 weeks of treatment, a reduction of 20.3 mmHg, 17.5 mmHg, and 12.1 mmHg was recorded in patients receiving baxdrostat at a dosage of 2 mg, 1 mg, 0.5 mg, respectively. The reduction was 9.4 mmHg in patients who received placebo. The reduction in DBP was also significantly greater in the 2 mg group (−14.3 mmHg), with a difference compared to placebo of −5.2 mmHg. No serious adverse events were linked to the drug or placebo and there were no cases of death or adrenocortical insufficiency. A slight increase in blood potassium levels occurred in some patients, but only three patients treated with baxdrostat experienced a significant increase in potassium levels, >6.0 mEq/l [74]. Phase 3 trials of baxdrostat are currently underway to evaluate the drug effects on a larger scale. Approximately 720 patients were enrolled, and treated with 1 mg or 2 mg baxdrostat or placebo once daily. The objective is to provide more significant data, also broadening the typology of patients, not just healthy individuals or small patient subgroups. Moreover, the phase 3 trials will evaluate the long-term effectiveness of baxdrostat in lowering aldosterone levels and its efficacy over an extended period [75]. The Target-HTN randomized trial evaluated the efficacy of another aldosterone synthase inhibitor, lorundrostat, in RH patients. An initial cohort of 163 participants with plasma renin activity less than or equal to 1.0 ng/mL/h and elevated plasma aldosterone levels (≥1.0 ng/dL) were enrolled, to which 37 participants with renin activity plasma concentration greater than 1.0 ng/mL/h were then added. They were randomized to receive one of five doses of lorundrostat in the initial cohort (12.5 mg, 50 mg, or 100 mg once daily or 12.5 mg or 25 mg twice daily) or placebo. In the second cohort, participants were then randomized 1:6 to receive lorundrostat 100 mg once daily or placebo. The primary endpoint was the change in SBP from baseline after 8 weeks of therapy. The trial showed that in patients with plasma renin activity ≤1.0 ng/mL/h, a reduction in SBP of 14.1 mmHg, 13.2 mmHg, and 6.9 mmHg was recorded, respectively, with a daily intake of lorundrostat 100 mg, 50 mg, and 12.5 mg. In patients subjected to placebo, however, an average reduction of 4.1 mmHg emerged. In patients receiving doses of 25 mg and 12.5 mg of lorundrostat twice daily, there was an observed reduction in SBP of 10.1 mmHg and 13.8 mmHg, respectively. Among patients with plasma renin activity greater than 1.0 ng/mL/h, however, treatment with lorundrostat 100 mg once daily reduced SBP by 11.4 mmHg. Similar to the treatment with baxdrostat, even with the use of lorundrostat an increase in serum potassium greater than 6.0 mEq/l occurred in only six participants, which was then corrected by reducing the dose or suspending the treatment [76]. The results of the Target-HTN trial indicate that the use of lorundrostat could have an important role in reducing BP in patients with uncontrolled HNT, although further studies are needed to confirm this effect [77].

### 5.3. NRP-1 Agonists

Among the causes of HNT and in particular of RH there is endothelial dysfunction, especially for impairment of nitric oxide vasodilation [78]. An endogenous analogue of arginine, asymmetric dimethylarginine (ADMA), inhibits the production of NO, thus hindering normal vascular homeostasis [78]. An increase in blood levels of ADMA has been observed in cerebral stroke, atherosclerosis, DM, CKD, HNT, and atherosclerosis [79]. Physiologically, ADMA is hydrolyzed by dimethylarginine dimethylaminohydrolase (DDAH) to citrulline and dimethylamines [80]. Two distinct isoforms of DDAH are known, the second of which makes only a small contribution to the accumulation of ADMA in tissues [80]. The most important isoform is DDAH1, of which there are some functional variants of the gene [81]. The expression of DDAHs is regulated by neuropilin-1 (NRP-1), a transmembrane glycoprotein, consisting of a large extracellular part and a short intracellular tail [82]. NRP-1 was first discovered as an antigen that binds to the A5 antibody, raised against neuronal cell surface proteins in the nervous system. It performs as an adhesion receptor in the nervous system and is implicated in axon guidance through its association with the semaphorin III family of proteins. It is also expressed in the heart, in stromal cells, in tumor cells, and in endothelial cells [83]. It was seen that in human umbilical vein endothelial cells (HUVECs) the absence of NRP-1 is associated with low levels and mRNA instability of DDAH1 but not DDAH2 [84]. Meanwhile, in mesenteric arteries and lung vascular endothelial cells of tamoxifen-inducible endothelial cell-specific NRP-1 knockout mice, decreased expression of DDAH1 and slightly increased expression of DDAH2 was exhibited [82]. NRP-1 regulates DDAH1 expression through posttranscriptional mechanisms [83,84]. Regarding the effect on blood pressure, it was seen that the endothelial cell-specific NRP-1 knockout mice did not show any significant change at the basal level, but after administration through osmotic minipumps of low-dose recombinant angiotensin II in both control and NRP-1 knockout mice it was seen that the increase in blood pressure levels was significantly greater in the second group [82]. Further studies are needed to clarify the exact role of NRP-1 in blood pressure regulation, but these first data suggest that drugs acting as NRP-1 agonists may represent another therapeutic option in patients with RH.

### 5.4. Zilebesiran

Zilebesiran (ALN-AGT01) is an RNA interference therapeutic agent for HNT. It inhibits hepatic synthesis of angiotensinogen (via reduction in messenger RNA for angiotensinogen in the liver) and consequently reduces circulating levels of angiotensin II [85]. The rationale behind its use is that although RAAS antagonists are commonly used to treat HNT, their effectiveness can be attenuated by compensatory reactivation of angiotensin and aldosterone escape [86]. To address this issue, dual RAAS blockade can be employed, albeit with a higher risk of adverse events such as hyperkalemia and worsening renal function, potentially related to excessive renal RAAS inhibition [87]. Therefore, upstream RAAS inhibition may be more effective, and specific silencing of hepatic angiotensinogen could limit adverse events associated with administration [88]. Additionally, the sustained pharmacodynamic effects of siRNAs allow for continuous BP reduction over 24 h and months, with semiannual or quarterly subcutaneous administration.

Recently, *The New England Journal of Medicine* published the results of a clinical trial evaluating the efficacy and safety of zilebesiran as a gene therapy for HNT [89]. This was a phase I study, randomized, double-blind, placebo-controlled, and conducted with multiple doses and an active comparator. The study was divided into four parts, each with different observation durations: part A which compared increasing single doses of zilebesiran (10, 25, 50, 100, 200, 400, 800 mg) with a placebo over 24 weeks; part B that compared a fixed single dose of the drug (800 mg) with a placebo under low and high salt diet conditions. The main objective was to evaluate the potential of dietary salt intake to modulate the antihypertensive effects of the drug; part C, originally planned as a multidose phase but removed from the protocol after an amendment; Part D, ongoing; part E, which evaluated a fixed single dose of zilebesiran (800 mg) in all patients. Those with SBP >120 mmHg at week 6 received daily co-administration of irbesartan 300 mg once a day for 2 weeks. The study population (n: 107) consisted of adults aged 18 to 65 years with mild-to-moderate HNT. Inclusion criteria involved mean seated SBP levels between 130 and 165 mmHg without antihypertensive medication for parts A, B, and D, and between 135 and 165 mmHg without treatment for part E. Exclusion criteria included secondary HNT, orthostatic hypotension, reduced renal function (eGFR < 60 mL/min/1.73 m^2^), diabetes mellitus, history of cardiovascular events, and intolerance to subcutaneous administration. The primary endpoint was the frequency of adverse events (observed for 12 months in parts A, B, and E; 18 months in part D). Secondary endpoints included changes from baseline in serum angiotensinogen levels and reductions in SBP and DBP measured via ambulatory monitoring over 24 h. The study observed dose-dependent reductions in angiotensinogen levels and BP, which were sustained up to 24 weeks. Common adverse events included mild and transient injection site reactions in five patients, with no cases of hypotension, hyperkalemia, or significant renal function reduction. BP variations could be reversed by a high-salt diet and were increased by co-administration of irbesartan. The results of the clinical trial appear to support the use of zilebesiran, as it is associated with consistent and lasting reductions in BP levels without an increased risk of adverse events. However, the analyzed sample size is small and not representative of the general population. Further studies are needed to evaluate the drug’s efficacy in specific target groups (e.g., elderly, diabetic, and chronic kidney disease patients). Additionally, the treatment’s ability to reduce hypertensive organ damage and impact cardiovascular risk in the medium and long term should be assessed. Recently, the results of the KARDIA-1 study were published [90]. This phase 2, randomized, double-blind study compared zilebesiran at increasing doses (150, 300, or 600 mg once every 6 months or 300 mg once every 3 months) with placebo (once every 3 months) over 6 months. Adults (n: 394) with mild-to-moderate HNT were randomized, defined as daytime mean SBP between 135 and 165 mmHg without treatment or during antihypertensive pharmacological washout. The primary endpoint was the change from baseline to month 3 in 24 h mean ambulatory SBP for each zilebesiran dose group compared to placebo. The effects on 24 h mean ambulatory systolic blood pressure were evident at the third month, with reductions ranging from 14.1 to 16.7 mm Hg compared to placebo, depending on the dose. Additionally, sustained reductions in angiotensinogen levels (exceeding 90%) were achieved at month 6 with doses of 300 mg or higher, suggesting potential efficacy at lower doses than observed in the phase 1 study [89]. These results support the possibility of administering zilebesiran subcutaneously every 6 months. This is particularly relevant because non-compliance with oral antihypertensive therapy remains a major factor influencing inadequate BP control in the general population. Furthermore, an ongoing study, KARDIA-2 (NCTO51O3332), will investigate the efficacy of zilebesiran as add-on therapy in patients with HNT inadequately controlled by olmesartan, amlodipine, or indapamide. A summary of these new drugs is provided in Table 2.

## 6. Perspectives

Arterial hypertension is one of the most widespread diseases in industrialized countries, affecting approximately 30% of the adult population, thus representing one of the major clinical problems of the modern era. The risk associated to the hypertensive diseases is more often underestimated. Even moderate elevation in arterial BP is associated with a reduction in life expectancy. It is also known as the “silent killer”, because it does not cause any symptoms and acts in the shadows, degenerating into severe complications, sometimes with fatal outcomes. Early identification of subjects at risk or already hypertensive is mandatory to reduce the burden of CV and non-CV complications as international guidelines recommend [1,3,10]. Despite the awareness of the risk associated with untreated HNT, in the last two decades pharmacological effort was missing with no new drugs available in clinical practice since the latest approval of aliskiren in 2007 by the FDA. Taking into account the total number of hypertensive patients not at target and the increasing number of patients affected by RH, the available pharmacological and non-pharmacological strategies seem insufficient. However, the latest randomized clinical trial specifically designed for hypertensive patients resulting in a final approval in clinical practice is dated 20 years ago [91]. Consequently, international guidelines have focused their new recommendations mainly on improving adherence in optimizing the available drugs as a combination strategy since the beginning and in considering interventional therapy such as renal denervation in a subset of patients if eligible [1,3,10]. Thus, there is a clear need of novelty in the field of hypertension in light of the tremendous advancement in basic research which has occurred in the last decade and new prespecified randomized clinical trials specifically evaluating new antihypertensive agents.

## 7. Conclusions

Hypertension remains the first and most modifiable risk factor worldwide. The scientific community has experienced a lack of novelty in this field lasting for almost 20 years. Research is ongoing but new and well-designed randomized clinical trials in the field are needed to improve daily management and reduce the current gaps. Meanwhile, physicians should continue to manage HNT and RH till help comes.

## Figures and Tables

**Table 1:** RH definitions.

	Definition
ESC	SBP ≥ 140 mmHg and DBP ≥ 90 mmHg despite lifestyle measures and OMT
AHA	Concurrent use of four or more antihypertensive drugs irrespective of BP levels

**Table 2:** Summary of the main characteristics of the new drugs.

Drug	Mechanism of Action	Trials	Effect on BP	Side Effect
Finerenone	selective nonsteroidal mineralocorticoid receptor antagonist (nsMRA)	FIDELIO-DKD FIGARO-DKD FIDELITY ARTS-ON	Lower effect on reducing office BP from baseline than spironolactone and eplerenone	Increase in K+ blood levels
Baxdrostat	selective enzymatic inhibition of aldosterone synthases	BRIGHT-HTN	Significant effect on reducing BP	Slight increase in K+ blood levels
Lorundrostat	selective enzymatic inhibition of aldosterone synthases	TARGET-HTN	Significant effect on reducing BP	Slight increase in K+ blood levels
NRP-1 agonists	neuropilin-1 acts indirectly on vascular tone through regulation of the expression of the enzyme DDAH which degrades the arginine analogue ADMA which inhibits NO synthesis	Preclinical studies on mice	Not known	Not known
Zilebesiran	an RNA interference therapeutic agent that inhibits hepatic synthesis of angiotensinogen and consequently reduces circulating levels of angiotensin II	Phase 1 trial KARDIA-1 KARDIA-2	Significant effect on reducing BP	Mild and transient injection site reactions

## Data Availability

The data from this manuscript are derived from publicly available published clinical trial and study results.
